# To Avoid or Not to Avoid: Cross‐Reactivity Between Fragrance and Common Botanicals

**DOI:** 10.1111/cod.70107

**Published:** 2026-02-06

**Authors:** Sarah Karels, Brailyn Weber, Sara Hylwa, Solveig Ophaug, Katherine Lee, Anne Neeley

**Affiliations:** ^1^ Department of Dermatology Park Nicollet Contact Dermatitis Clinic Minneapolis Minnesota USA; ^2^ University of Minnesota Medical School Minneapolis Minnesota USA; ^3^ University of North Dakota School of Medicine Grand Forks North Dakota USA; ^4^ Department of Dermatology University of Minnesota Minneapolis Minnesota USA

**Keywords:** allergic contact dermatitis, botanicals, contact allergy, cross‐reactivity, fragrance

## Abstract

**Background:**

Fragrance allergy is difficult to define, with thousands of known fragrance‐related compounds. Whether certain botanicals should be considered ‘fragrance’ and whether fragrance‐allergic patients should avoid these ingredients remains unclear, with minimal published data on the topic.

**Objectives:**

This study aimed to evaluate whether there is cross‐reactivity between fragrance and various botanicals commonly found in personal‐care products, such as aloe, coconut oil and shea butter, among others.

**Methods:**

A cohort of fragrance‐allergic patients was identified using data from 2038 patients patch‐tested at a tertiary referral centre from 2020–2024. Cross‐reactivity rates to botanicals commonly found in personal‐care products were calculated, and statistical significance was determined using Fisher's exact test. Cross‐reactivity was defined as > 10% reaction incidence.

**Results:**

In patients with fragrance allergy, there was no significant cross‐reactivity to any of the investigated botanical compounds.

**Conclusions:**

The low concomitant rates of reaction between fragrance and the investigated substances suggest that patients allergic to fragrance do not need to avoid certain common botanicals. Many products contain these compounds, including ones marked as ‘fragrance‐free’. Dermatologists can safely reassure fragrance‐allergic patients of the safety of many botanicals commonly used in personal‐care products.

## Graphical abstract

Among 356 fragrance‐allergic patients patch tested from 2020–2024, no clinically meaningful cross‐reactivity (> 10%) was observed between fragrance allergens and common non‐essential oil botanicals. These findings suggest fragrance‐allergic patients do not need to avoid botanicals such as aloe, coconut oil, or shea butter in fragrance‐free products.

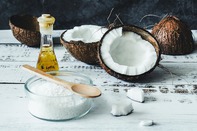

## 1 Introduction

Defining fragrance in the context of contact allergy remains a challenge. Too narrow a definition creates the potential for patients to face problematic exposures, while too broad a definition unnecessarily burdens patients with troublesome restrictions. Although many botanical compounds can be clearly and neatly categorised as fragrance, many others, such as aloe vera, shea butter, or coconut oil are more difficult to classify. Essential oils, which are distilled portions of specific botanicals, have been repeatedly shown to contain fragrance allergens and as such have been firmly categorised as ‘fragrance’ by previous studies [1]. These compounds include the common aromas of lavender oil, jasmine oil and tea tree oil, among others. Similar findings have been reported for floral compounds such as Compositae‐related allergens [2, 3]. Both Compositae allergens and these fragrant essential oils may contain terpenes, providing a plausible mechanism for true cross‐reactivity between these groups. The relationship between propolis, colophony, and fragrance has additionally been evaluated [4]. However, limited work has been performed examining non‐essential oil, non‐Compositae botanicals and their relationship with fragrance.

In this context, it is important to distinguish between true cross‐reactivity, where haptens share structural or chemical similarity, from concomitant sensitivity, where unrelated allergens co‐occur without a shared chemical basis. This distinction is particularly relevant when evaluating botanical ingredients that may co‐exist in personal care products but do not necessarily share allergenic determinants, as misclassification of non‐cross‐reacting botanicals impacts patient counselling and product avoidance.

One of the challenges in categorising these botanicals is the variability in fragrance allergen concentration between various parts of plants. As such, different botanical compounds may or may not contain the fragrance‐related compounds that trigger allergic reactions in fragrance‐sensitive individuals. There have been limited studies investigating the cross‐reactivity of these botanicals, but these have been limited in size and scope. For example, Simpson et al. found no reactions to olive oil or aloe vera in a cohort of 21 patients at high risk for botanical allergy [5]. Importantly, no large‐scale studies have broadly explored this topic. This study aims to address this gap by evaluating the cross‐reactivity between fragrance and non‐essential oil/non‐Compositae botanicals commonly used in personal‐care products. The botanicals included in this study were selected based on their frequent use in ‘fragrance‐free’ personal care products, their availability in purified forms for standardised patch testing, and their low irritancy potential. Where applicable, the tested materials correspond to commonly used International Nomenclature Cosmetic Ingredient (INCI) names; however, we acknowledge that variation in botanical sourcing, processing and labelling may limit direct extrapolation of patch‐test results to all commercially available formulations. Descriptions of the individual botanicals studied and their allergenicity as reported in published case reports are detailed below.

### 1.1 Aloe Vera

Aloe vera extract comes from the gel‐like centre pulp of the plant, which is made mostly of water and carbohydrates. Allergenic and irritant components can be found on the plant's exterior [6]. Reports of allergic contact dermatitis (ACD) and irritation to aloe have decreased over time as manufacturing has modernised to avoid contamination with the exterior [7].

### 1.2 Argan Oil

Argan oil refers to the unrefined oil from *Argania spinosa* kernels and is made mostly of triglycerides with < 1% composed of terpenes, phenols and sterols [8]. Several case reports describe contact allergy to argan oil [8, 9, 10, 11].

### 1.3 Candelilla Wax

Candelilla wax is extracted from the stalks of *Euphorbiacea* plants and is comprised of mostly hydrocarbons and large esters [12]. Though this plant family is known for producing an irritating, milky sap, Candelilla wax is refined in such a way that the hydrophilic sap does not precipitate into the final wax product [13]. Two case reports of contact allergy to candelilla wax have been published [12, 14].

### 1.4 Carnauba Wax

Carnauba wax is derived from the leaves of the *Copernicia prunifera* palm and is composed of esters, free alcohols, hydrocarbons and terpenes [15]. Two case reports of patch test positive contact allergy to carnauba wax have been published [16, 17].

### 1.5 Cocoa Butter

Cocoa butter comes from the seed of *Theobroma cacao* and is rich in fatty acids and tocopherols [18]. No case reports of cocoa butter contact allergy have been published.

### 1.6 Coconut Oil

Coconut oil is derived from the meat or milk of *Cocos nucifera* and contains triglycerides and phenols [19]. No case reports describing contact allergy to coconut oil have been published.

### 1.7 Green Tea Extract

Green tea extract comes from the fresh leaves of the *Camellia sinensis* plant and contains polyphenols, catechins, purine alkaloids, carbohydrates, lipids and vitamins [20]. No case reports of contact allergy specifically to green tea extract have been published; however, there has been documentation of patients reacting to tea leaves and blends during patch testing [21].

### 1.8 Jojoba Oil

Jojoba ‘oil’ is technically a liquid wax that is derived from the seeds of *Simmondsia chinensis* . It is primarily composed of wax esters, free fatty acids, hydrocarbons, alcohols, sterols, triglyceride esters and vitamins [19]. Two case reports of contact allergy to jojoba oil have been reported [22, 23].

### 1.9 Licorice Root Extract

Licorice root extract comes from the root of *Glycyrrhiza* sp. and is composed of saponins, flavonoids and polysaccharides [24]. At least 10 case reports of contact allergy due to licorice root extract currently exist [25].

### 1.10 Macadamia Nut Oil

Macadamia nut oil is cold pressed oil from the kernel of *Macadamia* tree species that contains fatty acids, tocopherols, sterols and phenols [26]. One case report of contact allergy to macadamia nut oil has been published [27].

### 1.11 Olive Oil

Olive oil is pressed from the flesh of the *Olea europaea* fruit and is composed of triglycerides, tocopherol, minerals and phenols [19]. Approximately 20 cases of contact allergy related to olive oil have been documented [28].

### 1.12 Shea Butter

Shea butter is the fat derived from the shea tree *Vitellaria paradoxa* (previously known as *Butyrospermum parkii*), which contains triglycerides, terpenes, tocopherols, phenols and sterols [29]. One case report documents a contact allergy to shea butter [30].

## 2 Methods

### 2.1 Patient Selection

This study included 2038 patients who underwent patch testing at the Park Nicollet Contact Dermatitis Clinic in Minneapolis, Minnesota between 2020 and 2024. It was declared exempt from review by the Health Partners Institute Institutional Review Board.

### 2.2 Patch Testing Process

Patients underwent comprehensive patch testing to the NACDG standard series (80 allergens, 2020) and in parallel to additional panels and allergens based on clinical history. As part of our clinic's practice, most patients are tested to an emulsifier series that contains the botanicals investigated. Finn Chambers (SmartPractice) or IQ Ultra Chambers (Chemotechnique) were used to affix patches to unaffected skin on patients' backs, upper arms and/or thighs. Reactions were interpreted on day 3 (48 h) and day 5 (96 h) or day 8 (168 h) (by board‐certified dermatologists according to International Contact Dermatitis Research Group (ICDRG) criteria) [31].

### 2.3 Inclusion

Patients were classified as ‘fragrance‐allergic’ if they exhibited a mild (1+) or greater positive reaction to at least one of the following fragrance screening mixes: Balsam of Peru, Fragrance Mix I, or Fragrance Mix II. Patients with a borderline (+/−) or greater reaction to the botanicals aloe vera, argan oil, candelilla wax, carnauba wax, cocoa butter, coconut oil, green tea extract, jojoba oil, licorice root extract, macadamia nut oil, olive oil, or shea butter were included for cross‐reaction comparisons, as these compounds had very low overall reaction rates. The source and method of testing for these compounds are outlined in Table 1.

**TABLE 1:** Source and testing method for botanical allergens included in study analysis.

Allergen	INCI name	Source	Testing method
Aloe vera	Aloe Barbadensis Leaf Juice	Nature's Oil 1X Aloe Vera Liquid; Streetsboro, Ohio	100% as‐is
Argan oil	Argania Spinosa Kernel Oil	Majestic Pure Cosmeceuticals Moroccan Argan Oil; Wilmington, Delaware	100% as‐is
Candelilla wax	Euphorbia Cerifera (Candelilla) Wax	Making Cosmetics Candelilla Wax; Redmond, Washington	50% in pet
Carnauba wax	Copernicia Cerifera (Carnauba) Wax	SpirZon Inc. Carnauba Wax; Durham, North Carolina	25% in pet
Cocoa butter	Theobroma Cacao (Cocoa) Seed Butter	Nature's Oil Naturally Refined Coca Butter; Streetsboro, Ohio	80% in pet
Coconut oil	Cocos Nucifera (Coconut) Oil	Happy Belly Organic Virgin Coconut Oil; Seattle, Washington	100% as‐is
Green tea extract	Camellia Sinensis Leaf Extract	Lotioncrafter Green Tea Extract; Eastsound, Washington	100% as‐is
Jojoba oil	Simmondsia Chinensis (Jojoba) Seed Oil	Aura Cacia Organic Jojoba Oil; Norway, Iowa	100% as‐is
Licorice root extract	Glycyrrhiza Glabra (Licorice) Root Extract	Starwest Botanicals Licorice Root Powder Organic; Sacramento, California	25% in pet
Macadamia nut oil	Macadamia Ternifolia Seed Oil	Lotioncrafter Macadamia Nut Oil; Eastsound, Washington	100% as‐is
Olive oil	Olea europaea (Olive) Fruit Oil	Market Pantry Extra Virgin Olive Oil; Minneapolis, Minnesota	100% as‐is
Shea butter	Butyrospermum Parkii (Shea) Butter	Nature's Oil Virgin (Organic) Unrefined Shea Butter; Streetsboro, Ohio	100% as‐is

*Note:* For each botanical, the commercial product used for patch testing is listed along with its INCI name, origin and the form/concentration tested. All products used featured the botanical in its pure form without added preservatives, fragrances, or other ingredients.

### 2.4 Data Analysis

Cross‐reactivity rates were calculated as the number of patients allergic to both a botanical *and* fragrance out of the number of patients allergic to fragrance only. A threshold of > 10% concomitant reactivity was set to define meaningful cross‐reactivity, based on the American Contact Dermatitis Society (ACDS) Contact Allergen Management Program (CAMP) guidelines [32]. Statistical significance was determined using Fisher's Exact test due to the small sample sizes in each subgroup, with a significance level of *α* = 0.05.

## 3 Results

Of the 2038 patients patch tested over this period, 356 (17.5%) were fragrance allergic. Though high, this prevalence is consistent with published data from large European surveillance networks reporting fragrance allergy rates from 12.7% to 17% and is likely further explained by the increased pre‐test probability for ACD in patients seen at our tertiary referral centre [33]. Cross‐reactivity analysis revealed that none of the included botanicals exhibited significant cross reactivity with fragrance. The calculated cross‐reactivity rates and their statistical significance are displayed in Table 2.

**TABLE 2:** Botanical co‐reactivity data and statistical significance.

Compound	Number of patients tested	Total reactions	Co‐reactions	Co‐reactivity rate	*p*
Coconut oil	1859	3	1	0.28%	0.44
Cocoa butter	1859	11	3	0.84%	0.42
Shea butter	1859	8	1	0.28%	> 0.99
Candelilla wax	1859	27	10	2.81%	0.02
Carnauba wax	1859	8	1	0.28%	> 0.99
Jojoba oil	1859	4	1	0.28%	0.54
Olive oil	1859	2	0	0.00%	> 0.99
Aloe vera	1859	23	9	2.53%	0.01
Argan oil	1859	3	0	0.00%	> 0.99
Sweet almond oil	1859	2	1	0.28%	0.32
Macadamia nut oil	1392	3	2	0.56%	0.08
Green tea	1009	6	1	0.28%	> 0.99
Licorice root	1009	20	8	2.25%	0.01

*Note:* Differences in the number of patients tested to specific compounds vary based on the point in time the substance was added to our clinic's standardised panels.

Although the cross‐reactivity rates for candelilla wax, aloe vera and licorice root reached statistical significance, their rates remained well below the 10% threshold of cross‐reaction significance utilised by the ACDS CAMP system.

## 4 Discussion

Our findings indicate that true chemical cross‐reactivity between fragrance allergens and the tested botanicals is likely minimal. Interestingly, the three botanicals that reached statistical significance (candelilla wax, aloe vera and licorice root) are not known to contain terpenes, unlike known fragrance allergens such as essential oils or Compositae‐related allergens. This supports the lack of a plausible chemical basis for true cross‐reactivity and suggests even these findings may relate to concomitant sensitivity.

Irritancy potential is an important consideration, particularly given the non‐standardised use of many of these botanicals in patch testing. As a tertiary referral centre with high rates of both allergic contact dermatitis and atopy, our patient population would be expected to demonstrate higher baseline rates of irritant and equivocal reactions. Despite this, the observed rates of borderline or positive reactions to the botanicals tested were very low and were therefore used as indirect evidence of low irritancy potential at the tested concentrations. In contrast, borderline (+/−) reactions to fragrance screening mixes were intentionally excluded from analysis due to the well‐recognised irritancy of these compounds. Although misclassification of irritant reactions as allergic responses can never be fully eliminated, the study design was structured to minimise this risk. Importantly, any residual misclassification would be expected to bias results towards higher apparent rates of concomitant reactivity; therefore, the consistently low observed rates further reinforce the conclusion that clinically meaningful cross‐reactivity is unlikely.

Defining cross‐reactors involves a fine balance of safety and restriction. If a patient who is allergic to a compound is not advised to avoid that compound's cross‐reactor, they may continue to have exposures that contribute to symptoms. Conversely, if patients are advised to avoid compounds that are not truly cross‐reactors to their allergens, they may be unnecessarily restricted in terms of safe product choices. Because fragrances are ubiquitous in products and manufacturers are not required to explicitly declare specific fragrances used, safe lists for allergic patients are often limiting, and consideration of inappropriate cross‐reactors only further restricts options. Additionally, many popular products marked as being ‘fragrance‐free’ utilise the botanical compounds analysed in this study but are otherwise free of fragrance allergens. At present, major allergen management programs in the United States either exclude these botanicals or provide the option to do so for fragrance‐allergic patients.

Unnecessary restriction of botanicals for fragrance‐allergic patients may also carry meaningful economic and practical consequences. Fragrance‐free products are often more expensive or less readily available than fragranced alternatives, as suitable options may need to be sourced online or from specialty retailers. Although availability of fragrance‐free products has improved in recent years, the least expensive personal‐care products, such as those sold in discount or dollar stores, frequently contain fragrance and therefore limit access for patients with financial constraints. If fragrance‐allergic individuals are additionally advised to avoid common botanicals without evidence of clinically meaningful cross‐reactivity, their range of safe, affordable over‐the‐counter options becomes even more restricted. This over‐avoidance may increase financial burden, contribute to frustration, and ultimately undermine adherence to allergen avoidance recommendations, with potential negative effects on dermatologic outcomes.

In contrast to the United States, the European Union has implemented expanded fragrance allergen labelling requirements, with legislation introduced in 2023 mandating explicit disclosure of additional fragrance substances when present above defined thresholds [34]. These regulatory differences may reduce the need for overly conservative avoidance strategies in European patients by improving transparency of fragrance content. In the U.S., however, the lack of mandatory disclosure of individual fragrance components continues to necessitate broader avoidance recommendations, making evidence‐based clarification of which non‐fragrance botanicals truly warrant exclusion particularly important. By reassuring fragrance‐allergic patients that botanicals such as coconut oil, aloe and shea butter are unlikely to cause contact reactions, dermatologists can help broaden patients' access to affordable and effective personal‐care products, ultimately improving quality of life and reducing the financial impact of fragrance allergy.

## 5 Limitations

This study was a retrospective, single‐centre investigation with a referral‐based population that may not be generalisable across all populations. Additional large‐scale, multi‐centre studies would be helpful in supporting this research. As noted in previous studies, botanical allergens and their preparation are not standardised, limiting accurate comparison [3]. Additionally, the tested botanicals may not perfectly correspond to all commercially available forms, as ingredient labelling using INCI names can vary based on plant part, processing, refinement, or added preservatives, creating uncertainty in extrapolating patch test results to every product formulation. The cross‐reactivity cutoff of > 10% was selected based on the ACDS CAMP guidelines, which established this threshold to balance inclusivity and exclusivity, rather than via a data‐driven metric [30]. Lastly, the low overall reaction counts for these compounds necessitate further research to expand the understanding of their cross‐reactions.

## 6 Conclusions

The findings of this study suggest that fragrance‐allergic patients do not need to avoid certain common botanicals, as cross‐reactivity between fragrance allergens and botanicals like aloe vera, coconut oil and shea butter is low and not clinically significant. Dermatologists can confidently recommend these botanicals in ‘fragrance‐free’ products, allowing fragrance‐allergic individuals to enjoy a broader range of personal care products.

## Author Contributions

**Sarah Karels:** conceptualization, investigation, writing – original draft, methodology, validation, project administration. **Brailyn Weber:** data curation, investigation, methodology, validation, writing − review & editing. **Sara Hylwa:** data curation, writing − review & editing. **Solveig Ophaug:** data curation, writing − review & editing. **Katherine Lee:** data curation, writing − review & editing. **Anne Neeley:** conceptualization, investigation, data curation, methodology, project administration, writing − review & editing.

## Funding

The authors have nothing to report.

## Ethics Statement

This study was determined to be exempt from review by the HealthPartners Institute IRB.

## Conflicts of Interest

Sara Hylwa, MD is a paid speaker for Contact Dermatitis Institute. The other authors declare no conflicts of interest.

## Data Availability

The data that support the findings of this study are available from the corresponding author upon reasonable request.
